# Supplementary material for: The Association of Gene Variants in the Vitamin D Metabolic Pathway and Its Interaction with Vitamin D on Gestational Diabetes Mellitus: A Prospective Cohort Study
Source: Nutrients. 2021 Nov 24;13(12):4220. doi: 10.3390/nu13124220 (PMC8706628; doi:10.3390/nu13124220)
Supplement: Supplementary file 1 [file nutrients-13-04220-s001.zip › nutrients-1432438-supplementary.pdf]

Supplementary Table S1. Baseline characteristics of pregnant women with different GDM subtypes

Variables	GDM subtype 1 *n* = 144	GDM subtype 2 *n* = 300	GDM subtype 3 *n* = 69	*P*
**Age, yrs**	28.4±3.6	30.0±4.2	30.4±3.6	<0.0001
**Prepregnancy BMI (kg/m^2)**	21.4±2.9	21.0±3.0	22.7±2.7	<0.0001
**25(OH)D at T1 (ng/mL)** [a]	17.6±6.8	20.3±9.6	19.9±6.7	0.0180
**25(OH)D at T2 (ng/mL)** [b]	20.3±8.8	27.7±11.1	21.3±9.6	<0.0001
**VitD deficiency at T1** [a]	97 (69.3%)	171 (58.6%)	34 (52.3%)	0.0332
**VitD deficiency at T2** [b]	68 (61.8%)	38 (25.7%)	21 (50.0%)	<0.0001
**OGTT season**				<0.0001
Summer/fall	37 (25.7%)	178 (59.3%)	17 (24.6%)	
Winter/spring	107 (74.3%)	122 (40.7%)	52 (75.4%)	
**Educational level**				0.0497
≤ High school	46 (31.9%)	85 (28.3%)	30 (43.5%)	
> High school	98 (68.1%)	215 (71.7%)	39 (56.5%)	
**Income per capita, yuan**				0.1494
<30,000	20 (13.89%)	25 (8.33%)	3 (4.35%)	
≥30,000	106 (73.61%)	216 (72%)	56 (81.16%)	
Not sure	9 (6.25%)	33 (11%)	6 (8.7%)	
Unknown	9 (6.25%)	26 (8.67%)	4 (5.8%)	
**Planned pregnancy**				0.2839
No	35 (24.31%)	88 (29.33%)	23 (33.33%)	
Yes	102 (70.83%)	186 (62%)	42 (60.87%)	
Unknown	7 (4.86%)	26 (8.67%)	4 (5.8%)	
**Marital status**				0.0011[*]
Not married	4 (2.78%)	7 (2.33%)	1 (1.45%)	

Married	133 (92.36%)	268 (89.33%)	63 (91.3%)	
Unknown	7 (4.86%)	25 (8.33%)	5 (7.25%)	
**VitD supplement**				0.4831
0/week	56 (38.89%)	92 (30.67%)	24 (34.78%)	
>0/week	79 (54.86%)	181 (60.33%)	39 (56.52%)	
Unknown	9 (6.25%)	27 (9%)	6 (8.7%)	
**Primiparity**				0.0002
No	25 (17.36%)	81 (27%)	26 (37.68%)	
Yes	116 (80.56%)	189 (63%)	37 (53.62%)	
Unknown	3 (2.08%)	30 (10%)	6 (8.7%)	
**Physical exercise**				0.9600
0/week	110 (76.39%)	226 (75.33%)	55 (79.71%)	
>0/week	22 (15.28%)	48 (16%)	9 (13.04%)	
Unknown	12 (8.33%)	26 (8.67%)	5 (7.25%)	

Abbreviation: GDM, gestational diabetes mellitus; VitD, vitamin D; T1, first trimester; T2, second trimester; OGTT, oral glucose tolerance test.

[a] n = 497, [b] n = 300

[*]Fisher's exact test

Supplementary Table S2. Association of SNPs and its interaction with 25(OH)D concentrations at T1 and T2 on three time-point plasma glucose level of OGTT [*].

SNPs	Genotypes	*n*	FBG (mmol/L)			PG1H (mmol/L)			PG2H (mmol/L)		
			Mean ±SD (mmol/L)	β (SE)	*P*	Mean ±SD (mmol/L)	β (SE)	*P*	Mean ±SD (mmol/L)	β (SE)	*P*
**VitD-related SNPs**											
CYP24A1											
rs2209314	TT	770	4.55±0.47	Ref	—	7.75±1.65	Ref	—	6.93±1.40	Ref	—
	CT	1039	4.53±0.45	-0.01 (0.02)	0.4607	7.83±1.62	0.10 (0.08)	0.1992	6.92±1.37	-0.00 (0.06)	0.9439 [†]
	CC	335	4.53±0.44	-0.01 (0.03)	0.6233	7.82±1.64	0.07 (0.10)	0.4737	6.92±1.23	-0.01 (0.09)	0.9385
CYP3A4											
rs2242480	CC	1229	4.54±0.45	Ref	—	7.79±1.61	Ref	—	6.93±1.37	Ref	—
	CT	790	4.53±0.47	0.02 (0.02)	0.3588	7.79±1.67	-0.02 (0.07)	0.8164 [†]	6.89±1.35	-0.08 (0.06)	0.1617
	TT	125	4.52±0.39	0.01 (0.04)	0.8962	7.91±1.65	0.12 (0.15)	0.4050	6.98±1.41	0.03 (0.12)	0.7956
GC											
rs1155563	TT	761	4.53±0.46	Ref	—	7.75±1.61	Ref	—	6.91±1.36	Ref	—
	TC	1019	4.54±0.45	0.01 (0.02)	0.6546	7.79±1.67	0.06 (0.08)	0.4289	6.91±1.35	0.02 (0.06)	0.7995
	CC	362	4.54±0.48	0.01 (0.03)	0.7430	7.90±1.56	0.12 (0.10)	0.2580	6.96±1.41	0.03 (0.08)	0.7598
rs16846876	AA	1017	4.53±0.46	Ref	—	7.75±1.65	Ref	—	6.88±1.35	Ref	—
	AT	899	4.54±0.46	0.00 (0.02)	0.9360	7.85±1.59	0.08 (0.07)	0.2929	6.96±1.37	0.05 (0.06)	0.3846
	TT	231	4.55±0.45	0.02 (0.03)	0.5963	7.77±1.73	-0.02 (0.12)	0.8918	6.90±1.41	-0.01 (0.10)	0.9086 [†]
rs17467825	AA	1008	4.52±0.45	Ref	—	7.78±1.62	Ref	—	6.90±1.34	Ref	—
	GA	909	4.55±0.46	0.01 (0.02)	0.5559	7.81±1.63	0.02 (0.07)	0.7811	6.95±1.38	0.05 (0.06)	0.4486
	GG	234	4.55±0.45	0.01 (0.03)	0.6360	7.81±1.69	-0.01 (0.12)	0.9115	6.91±1.40	-0.01 (0.10)	0.9414
rs2282679	TT	1009	4.52±0.45	Ref	—	7.79±1.62	Ref	—	6.91±1.33	Ref	—
	GT	899	4.55±0.46	0.01 (0.02)	0.7194	7.81±1.63	0.02 (0.07)	0.7747	6.94±1.39	0.04 (0.06)	0.5404

	GG	241	4.55±0.45	0.01 (0.03)	0.7744	7.79±1.69	-0.04 (0.11)	0.7393	6.91±1.39	-0.02 (0.09)	0.8468
rs2298849	AA	894	4.55±0.45	Ref	—	7.83±1.60	Ref	—	6.94±1.34	Ref	—
	GA	960	4.52±0.46	-0.02 (0.02)	0.3963	7.79±1.69	-0.02 (0.07)	0.8155 [†]	6.92±1.39	-0.00 (0.06)	0.9476 [†]
	GG	299	4.53±0.44	-0.03 (0.03)	0.3182	7.69±1.57	-0.16 (0.11)	0.1451	6.87±1.35	-0.08 (0.09)	0.3667
rs2298850	GG	982	4.52±0.45	Ref	—	7.78±1.63	Ref	—	6.90±1.33	Ref	—
	CG	911	4.55±0.46	0.01 (0.02)	0.6255	7.81±1.64	0.02 (0.07)	0.7636	6.94±1.39	0.04 (0.06)	0.5214
	CC	240	4.54±0.45	0.01 (0.03)	0.8547	7.77±1.68	-0.05 (0.11)	0.6448	6.90±1.39	-0.02 (0.09)	0.8470
rs3755967	CC	1005	4.52±0.45	Ref	—	7.78±1.62	Ref	—	6.90±1.33	Ref	—
	CT	907	4.55±0.46	0.01 (0.02)	0.6228	7.81±1.64	0.03 (0.07)	0.6711	6.94±1.39	0.04 (0.06)	0.4980
	TT	241	4.55±0.45	0.01 (0.03)	0.7422	7.79±1.69	-0.03 (0.11)	0.7815	6.91±1.39	-0.02 (0.09)	0.8635
rs4588	GG	994	4.52±0.46	Ref	—	7.79±1.62	Ref	—	6.91±1.33	Ref	—
	GT	909	4.55±0.46	0.01 (0.02)	0.7144	7.80±1.64	0.01 (0.07)	0.8886	6.94±1.39	0.03 (0.06)	0.6419
	TT	241	4.54±0.45	0.00 (0.03)	0.9165	7.78±1.69	-0.05 (0.11)	0.6719	6.90±1.39	-0.03 (0.09)	0.7398
rs7041	AA	1162	4.55±0.46	Ref	—	7.78±1.64	Ref	—	6.91±1.36	Ref	—
	CA	826	4.53±0.45	-0.02 (0.02)	0.2791	7.80±1.61	0.02 (0.07)	0.7835	6.92±1.37	0.00 (0.06)	0.9442
	CC	162	4.52±0.49	0.02 (0.03)	0.5486	7.85±1.70	0.10 (0.13)	0.4754	6.98±1.30	0.06 (0.11)	0.5571
LRP2											
rs10210408	CC	703	4.53±0.43	Ref	—	7.89±1.66	Ref	—	7.03±1.39	Ref	—
	TC	1065	4.53±0.47	0.00 (0.02)	0.9225	7.71±1.64	-0.19 (0.08)	0.0119	6.84±1.34	-0.21 (0.06)	0.0012
	TT	385	4.55±0.48	0.03 (0.03)	0.3012	7.84±1.56	-0.03 (0.10)	0.7792	6.94±1.35	-0.06 (0.08)	0.4514
VDR											
rs10783219	AA	809	4.53±0.45	Ref	—	7.72±1.57	Ref	—	6.88±1.31	Ref	—
	TA	1010	4.54±0.45	0.00 (0.02)	0.8998	7.82±1.64	0.10 (0.07)	0.1741	6.94±1.40	0.08 (0.06)	0.2237
	TT	332	4.54±0.49	-0.00 (0.03)	0.9873	7.93±1.76	0.24 (0.10)	0.0212	6.96±1.36	0.09 (0.09)	0.2890
**GDM-related SNPs**											
CDKAL1											
rs7754840	GG	635	4.49±0.46	Ref	—	7.72±1.61	Ref	—	7.00±1.32	Ref	—

	GC	820	4.50±0.42	0.01 (0.02)	0.5145	7.85±1.55	0.12 (0.08)	0.1541	6.95±1.38	-0.05 (0.07)	0.4358
	CC	264	4.50±0.46	0.02 (0.03)	0.4053	7.90±1.75	0.20 (0.11)	0.0766 †	7.10±1.40	0.13 (0.10)	0.1803
rs7754840	GC/GG	1455	4.49±0.44	Ref	—	7.79±1.58	Ref	—	6.97±1.35	Ref	—
	CC	264	4.50±0.46	0.01 (0.03)	0.6553	7.90±1.75	0.15 (0.10)	0.1432	7.10±1.40	0.19 (0.08)	0.0211
IGF2BP2											
rs1470579	AA	966	4.50±0.44	Ref	—	7.79±1.56	Ref	—	7.00±1.36	Ref	—
	CA	664	4.49±0.43	-0.01 (0.02)	0.7562	7.82±1.68	0.04 (0.08)	0.6519	6.97±1.37	-0.02 (0.07)	0.7266
	CC	89	4.43±0.47	-0.07 (0.04)	0.0870	7.87±1.54	0.04 (0.17)	0.8002	7.00±1.25	-0.02 (0.14)	0.8833
MTNR1B											
rs10830962	CC	572	4.48±0.42	Ref	—	7.58±1.54	Ref	—	6.88±1.31	Ref	—
	GC	850	4.49±0.46	0.03 (0.02)	0.1968	7.84±1.64	0.26 (0.08)	0.0019	7.02±1.40	0.13 (0.07)	0.0601 †
	GG	297	4.52±0.44	0.08 (0.03)	0.0041	8.16±1.56	0.61 (0.11)	<.0001	7.11±1.33	0.24 (0.09)	0.0113
PRKCE											
rs11682804	GG	839	4.47±0.44	Ref	—	7.73±1.57	Ref	—	6.91±1.34	Ref	—
	AG	745	4.51±0.44	0.04 (0.02)	0.0254	7.92±1.63	0.17 (0.08)	0.0335	7.07±1.38	0.13 (0.07)	0.0440
	AA	138	4.53±0.45	0.05 (0.04)	0.1504	7.66±1.61	-0.07 (0.14)	0.6070	7.02±1.36	0.09 (0.12)	0.4547

Abbreviations: VitD, vitamin D; OGTT, oral glucose tolerance test; FBG, fasting blood glucose; PG1H, 1h-postprandial glucose; PG2H, 2h-postprandial glucose.

* Adjusted for maternal age, prepregnancy BMI, parity, educational level, income, physical exercise and OGTT season.

† *P* value of the interaction term SNPs * VitD level at first trimester < 0.05.

Supplementary Table S3. Association of Gc isoforms and different time-point plasma glucose level of OGTT

**Gc subtypes**	***n***	**FBG**			**PG1H**			**PG2H**		
		**Mean ± SD (mmol/L)**	**β (SE)**	***P***	**Mean ± SD (mmol/L)**	**β (SE)**	***P***	**Mean ± SD (mmol/L)**	**β (SE)**	***P***
**Total Participants ***										
1f/1f	378	4.54±0.44	Ref	—	7.81±1.61	Ref	—	6.97±1.34	Ref	—
1f/2	540	4.55±0.47	-0.003 (0.027)	0.912	7.77±1.65	-0.018 (0.107)	0.863	6.89±1.37	-0.047 (0.088)	0.593
2/2	241	4.54±0.45	-0.010 (0.034)	0.767	7.78±1.69	-0.054 (0.132)	0.679	6.90±1.39	-0.075 (0.108)	0.486
1s/1f	455	4.51±0.45	-0.034 (0.028)	0.239	7.75±1.60	-0.039 (0.111)	0.725	6.84±1.33	-0.099 (0.091)	0.277
1s/2	369	4.54±0.45	-0.011 (0.030)	0.712	7.86±1.63	0.038 (0.117)	0.747	7.00±1.42	0.029 (0.096)	0.763
1s/1s	161	4.52±0.49	0.014 (0.039)	0.725	7.85±1.71	0.074 (0.150)	0.620	6.97±1.29	0.008 (0.123)	0.946
**BMI < 24 ⁋**										
1f/1f	334	4.53±0.43	Ref	—	7.81±1.60	Ref	—	6.99±1.35	Ref	—
1f/2	471	4.52±0.44	-0.021 (0.028)	0.459	7.68±1.60	-0.077 (0.114)	0.496	6.87±1.40	-0.074 (0.094)	0.429
2/2	200	4.51±0.42	-0.024 (0.035)	0.490	7.61±1.69	-0.151 (0.142)	0.288	6.78±1.36	-0.173 (0.117)	0.139
1s/1f	408	4.49±0.44	-0.047 (0.029)	0.105	7.68±1.58	-0.089 (0.117)	0.449	6.79±1.31	-0.164 (0.096)	0.089
1s/2	325	4.54±0.45	-0.009 (0.031)	0.774	7.77±1.63	-0.031 (0.124)	0.799	6.92±1.42	-0.050 (0.102)	0.625
1s/1s	144	4.51±0.46	-0.002 (0.039)	0.957	7.74±1.69	-0.035 (0.158)	0.827	6.94±1.24	-0.041 (0.130)	0.753
**BMI ≥ 24 ⁋**										
1f/1f	44	4.59±0.52	Ref	—	7.86±1.73	Ref	—	6.78±1.27	Ref	—
1f/2	69	4.75±0.61	0.153 (0.098)	0.120	8.37±1.81	0.560 (0.323)	0.084	7.06±1.10	0.200 (0.258)	0.438
2/2	41	4.69±0.53	0.120 (0.111)	0.281	8.64±1.38	0.741 (0.364)	**0.043**	7.50±1.39	0.707 (0.291)	**0.016**
1s/1f	47	4.69±0.52	0.072 (0.108)	0.506	8.32±1.65	0.355 (0.353)	0.316	7.31±1.43	0.462 (0.282)	0.103
1s/2	44	4.58±0.45	0.019 (0.110)	0.866	8.52±1.51	0.776 (0.360)	**0.032**	7.57±1.32	0.582 (0.288)	**0.044**
1s/1s	17	4.66±0.70	0.166 (0.145)	0.251	8.83±1.57	1.100 (0.474)	**0.021**	7.20±1.68	0.346 (0.379)	0.362

	P_{trend} = 0.995	P_{trend} = **0.031**	P_{trend} = **0.037**

Abbreviations: OGTT, oral glucose tolerance test; FBG, fasting blood glucose; PG1H, 1h-postprandial glucose; PG2H, 2h-postprandial glucose.

* Adjusted for maternal age, prepregnancy BMI, parity, educational level, income, physical exercise and OGTT season.

⁋ Adjusted for maternal age, parity, educational level, income, physical exercise and OGTT season.

Supplementary Table S4. Association of Gc isoforms with GDM. and GDM. subtypes among women with different prepregnancy BMI *.

Gc isoforms	*n*	GDM. †		GDM. subtype 1 ‡		GDM. subtype 2 ‡		GDM. subtype 3 ‡	
		case (%)	OR (95% CI)	case (%)	OR (95% CI)	case (%)	OR (95% CI)	case (%)	OR (95% CI)
**BMI < 24**									
1f/1f	334	76 (22.8)	Ref	21 (6.3)	Ref	42 (12.6)	Ref	13 (3.9)	Ref
1f/2	471	105 (22.3)	0.99 (0.71-1.40)	30 (6.4)	0.92 (0.51-1.66)	67 (14.2)	1.19 (0.78-1.83)	8 (1.7)	0.44 (0.18-1.10)
2/2	200	44 (22.0)	0.98 (0.64-1.50)	13 (6.5)	0.98 (0.47-2.04)	27 (13.5)	1.16 (0.68-1.97)	4 (2.0)	0.46 (0.14-1.48)
1s/1f	408	83 (20.3)	0.88 (0.62-1.26)	22 (5.4)	0.79 (0.42-1.48)	50 (12.3)	1.01 (0.64-1.58)	11 (2.7)	0.66 (0.29-1.54)
1s/2	325	77 (23.7)	1.05 (0.73-1.52)	24 (7.4)	1.10 (0.59-2.06)	47 (14.5)	1.22 (0.77-1.93)	6 (1.9)	0.45 (0.17-1.24)
1s/1s	144	32 (22.2)	1.00 (0.62-1.62)	7 (4.9)	0.79 (0.32-1.95)	22 (15.3)	1.24 (0.70-2.20)	3 (2.1)	0.62 (0.17-2.30)
**BMI ≥ 24**									
1f/1f	44	7 (15.9)	Ref	3 (6.8)	Ref	1 (2.3)	Ref	3 (6.8)	Ref
1f/2	69	24 (34.8)	2.81 (1.06-7.50) §	9 (13.0)	2.96 (0.67-13.02)	8 (11.6)	6.11 (0.71-52.29)	7 (10.1)	1.99 (0.43-9.11)
2/2	41	15 (36.6)	3.09 (1.04-9.21) §	3 (7.3)	1.10 (0.17-7.30)	8 (19.5)	11.01 (1.24-98.11) §	4 (9.8)	2.38 (0.41-13.81)
1s/1f	47	20 (42.6)	3.54 (1.26-9.94) §	7 (14.9)	3.84 (0.80-18.41)	9 (19.2)	11.00 (1.28-94.90) §	4 (8.5)	1.28 (0.23-7.15)
1s/2	44	20 (45.5)	4.18 (1.46-11.94) §	4 (9.1)	2.48 (0.45-13.66)	12 (27.3)	15.23 (1.79-129.64) §	4 (9.1)	2.17 (0.40-11.80)
1s/1s	17	8 (47.1)	5.62 (1.53-20.57) §	1 (5.9)	1.56 (0.13-18.37)	5 (29.4)	20.45 (2.01-207.97) §	2 (11.8)	4.46 (0.53-37.37)
			$P_{trend} = 0.0046$		$P_{trend} = 0.4177$		$P_{trend} = 0.0011$		$P_{trend} = 0.3643$

Abbreviations: GDM., gestational diabetes mellitus; subtype 1, elevated fasting glucose and normal postload glucose; subtype 2, normal fasting glucose and elevated postload glucose; subtype 3, elevated fasting and postload glucose.

* Adjusted for maternal age, parity, educational level, income, physical exercise and OGTT season.

† Binomial logistic regression model; ‡ Multinomial logistic regression model.

§ $P < 0.05$.
